# 98 Plasma Extracellular Vesicles Released After Sever Burn Injury Modulate Macrophage Function

**DOI:** 10.1093/jbcr/irac012.101

**Published:** 2022-03-23

**Authors:** Micah L Willis, Cressida Mahung, Shannon Wallet, Leon Coleman, Robert Maile

**Affiliations:** University of North Carolina Chapel Hill, Durham, North Carolina; University of North Carolina at Chapel Hill, Washington, District of Columbia; University of North Carolina at Chapel Hill, Chapel Hill, North Carolina; UNC-CH, Chapel Hill, North Carolina; UNC, Chapel Hill, North Carolina

## Abstract

**Introduction:**

Extracellular microvesicles (MVs) have emerged as key regulators of immune function across multiple diseases and potential biomarkers. Severe burn injury is a devastating trauma with significant immune dysfunction that results in an ~12% mortality rate due to sepsis-induced organ failure, pneumonia, and other infections. Severe burn causes a biphasic immune response: an early (0-72 hrs) hyper-inflammatory state, with release of pro-inflammatory damage-associated molecular pattern molecules (DAMPs), such as HMGB1, and cytokines (e.g.IL-1b), followed by an immunosuppressive state (1-2 weeks post injury), associated with increased susceptibility to life-threatening infections. We have reported that early after severe burn injury HMGB1 and IL-1b are enriched in plasma microvesicles (MVs), suggesting a role for MVs in post-burn immune activation. Here we tested the impact of MVs isolated after burn injury on phenotypic and functional consequences *in vivo* and *in vitro* using adoptive MV transfers.

**Methods:**

We then assessed if the cargo of MVs following burn injury in humans could predict length of hospital stay. MVs isolated early from mice that underwent a 20% total body surface area (TBSA) burn injury (burn MVs) caused similar cytokine responses in naïve mice to those seen in burned mice early after injury. Burn MVs transferred to RAW264.7 macrophages caused similar functional (i.e. cytokine secretion) and genetic changes (measured by NanoString^TM^) seen with their associated phase of post-burn immune dysfunction.

**Results:**

Burn MVs isolated early (24h) induced MCP-1, IL-12p70 and IFNg, while MVs isolated later (1 and 2 weeks) blunted RAW proinflammatory responses to bacterial endotoxin (LPS). Unbiased LC-MS / MS proteomic analysis of early EVS (< 72 h post-injury) showed similarities in human and mice.

**Conclusions:**

In our sample of large burn injury, EV SAA1 and CRP correlated with TBSA injury in both sexes and were correlated with length of hospital stay in women.

